# Supplementary material for: Biochemical basis for the regulation of biosynthesis of antiparasitics by bacterial hormones
Source: eLife. 2020 Jun 8;9:e57824. doi: 10.7554/eLife.57824 (PMC7347384; doi:10.7554/eLife.57824)
Supplement: Supplementary file 1. [file elife-57824-supp1.docx]

| **Key Resources Table** | | | | |
| --- | --- | --- | --- | --- |
| **Reagent type (species) or resource** | **Designation** | **Source or reference** | **Identifiers** | **Additional information** |
| Strain, strain background (*Streptomyces avermitilis)* | *Streptomyces avermitilis* | ATCC 31267  NRRL 8165 | *S. avermitilis* |  |
| Gene (*Streptomyces avermitilis*) | AvaR1 |  | Uniprot: Q82H41 |  |
| Strain, strain background ( *Streptomyces phaeochromogenes*) | *Streptomyces phaeochromogenes* | NRRL, B-3010 | *S. phaeochromogenes* |  |
| Strain, strain background (*Streptomyces viridosporus*) | *Streptomyces viridosporus* | NRRL, ISP-5243 | *S. viridosporus* |  |
| Strain, strain background (*Escherichia coli* DH5α) | DH5α | Thermo Fisher | DH5 α | Plasmid upkeep |
| Strain, strain background (*Escherichia coli* Rosetta (DE3)) | *E. coli* Rosetta (DE3) | Novagen | Rosetta | Protein expression |
| Transformed construct *Escherichia coli* Rosetta (DE3) | pET28-MBP-AvaR1 | This paper | pET28-MBP-AvaR1 | Plasmid may be obtained from author |
| Recombinant protein | MBP-AvaR1 | This paper | MBP-AvaR1 | Plasmid may be obtained from author |
| Recombinant protein | MBP-SeMet-AvaR1 | This paper | MBP-SeMet-AvaR1 | Plasmid may be obtained from author |
| Chemical compound, drug | Selenomethionine | Fisher Scientific, 56-150-51GM | SeMet | Used in SeMet-AvaR1 generation |
| Chemical compound, drug | Kanamycin monosulfate | GoldBio, K-120-50 | Kanamycin |  |
| Chemical compound, drug | Chloramphenicol | GoldBio, C-105-5 | Chloramphenicol |  |
| Chemical compound, drug | Isopropyl β- d-1-thiogalactopyranoside | GoldBio, I2481C100 | IPTG |  |
| Chemical compound, drug | NaCl | Fisher scientific, 18-606-411 | NaCl | Protein purification buffer |
| Chemical compound, drug | KCl | Fisher scientific, 18-605-511 | KCl | Size exclusion buffer |
| Chemical compound, drug | Tris Base | Fisher scientific, BP152-1 | Tris | Protein purification buffer |
| Chemical compound, drug | HEPES | Fisher scientific, AAJ1692622 | HEPES | Size exclusion buffer |
| Chemical compound, drug | Imidazole | Fisher scientific, AC122020050 | Imidazole | Nickel column elution |
| Chemical compound, drug | Glycerol | Fisher scientific, G33-500 | Glycerol | Cell lysis buffer, cell cryopreservation |
| Chemical compound, drug | Sodium acetate trihydrate | Fisher scientific, S209-500 | Sodium acetate trihydrate | Crystallization condition reagent |
| Chemical compound, drug | Ethylene glycol | Fisher scientific, E178-500 | Ethylene glycol | Crystal cryoprotectant |
| Chemical compound, drug | KBr | Fisher scientific, AC206390250 | KBr | Crystallization reagent |
| Chemical compound, drug | Poly(ethylene glycol) monomethylether average molecular weight 2000 | Fisher scientific, NC1745431 | PEG 2000 MME | Crystallization reagent |
| Chemical compound, drug | Sodium citrate tribasic dihydrate | Fisher scientific, S279-500 | Sodium citrate tribasic dihydrate | Crystallization reagent |
| Chemical compound, drug | Tert-butanol | Fisher scientific, A401-500 | Tert-butanol | Crystallization reagent |
| Chemical compound, drug | Lithium sulfate | Fisher scientific, AAA1041022 | Lithium Sulfate | Crystallization reagent |
| Chemical compound, drug | Poly(ethylene glycol) Average MW 1000 | Fisher scientific, AC192250010 | PEG 1000 | Crystallization reagent |
| Chemical compound, drug | Poly(ethylene glycol) Average MW 3350 | Fisher scientific, P146-3 | PEG 3350 | Crystallization condition reagent |
| Sequence-based reagent | Bovine Thrombin | Fisher Scientific, ICN15416301 | Thrombin | Removal of expression tag |
| Sequence-based reagent | BamHI-HF | New England Biolabs, R3136S | BamHI | Gene cloning |
| Sequence-based reagent | XhoI | New England Biolabs, R0146S | XhoI | Gene cloning |
| Sequence-based reagent | SalI | New England Biolabs, R0138S | SalI | Gene cloning |
| Sequence-based reagent | BglII | New England Biolabs, R0144S | BglII | Gene cloning |
| Software, algorithm | EFI-EST | <https://efi.igb.illinois.edu/> |  | Used to create SSN |
| Software, algorithm | EFI-GNT | <https://efi.igb.illinois.edu/> |  | Used to create GNN |
| Other | GE Healthcare HisTrap HP | GE Healthcare | 5 mL HiTrap Ni Chelating column | Protein purification |
| Other | SuperDex HiLoad 75 16/60 column | GE Healthcare | Size exclusion column | Isolation of protein dimers |
| Sequence-based reagent | acoARE_Pal2-1-5’GC | This paper | oligonucleotide | GCAAGATACGTACTGCAGTACGTATCTTGC |
| Other | (4S,10R)-10-hydroxy-10-methyl-9-oxo-dodec-2-en-1,4-olide | This paper | Avenolide | Synthesis is described in this work |
| Chemical compound, drug | LB broth | Fisher Scientific, BP1426-2 | LB | Cocrystallization, ITC, and culture treatment |
| Chemical compound, drug | Gibco Difco Yeast extract | Fisher scientific, DF210929 | Yeast extract |  |
| Chemical compound, drug | Gibco Bacto Malt extract | Fisher scientific, DF0186-17-7 | Malt extract | Used to make ISP2 media |
| Chemical compound, drug | Dextrose | Fisher scientific, D16-500 |  | Used to make ISP2 media |
| Software, algorithm | Phenix | <https://www.phenix-online.org/> |  | Used to make ISP2 media |
| Software, algorithm | CCP4 | <http://www.ccp4.ac.uk/> |  | Phasing, initial model building |
| Software, algorithm | Cytoscape | 30 |  | Structure Refinement |
| Software, algorithm | AutoPROC | 28 |  | SSN and GNN visualization |
| Software, algorithm | COOT | 29 |  | Crystallographic data processing |
| Software, algorithm | UCSD Chimera | <https://www.cgl.ucsf.edu/chimera/> |  | Manual refinement and ligand fitting |
| Other | Minispin columns | Syd Labs |  | Structure visualization |
| Commercial kit | UltraClean Microbial DNA Isolation Kit | Mo Bio |  | Plasmid purification |
| Commercial kit | Index screen | Hampton Research |  | Genomic DNA isolation |
| Other | 96-2 well INTELLI-PLATE Shallow well screening trays | Hampton Research,  HR3-164 | Screening tray | Initial crystallization condition screen |
| Other | 48 well trays | Fisher Scientific | Optimization tray | Screening of initial crystallization conditions |
| Commercial kit | Additive Screen | Hampton Research,  HR2-428 | Additives for crystal optimization | Optimization of crystallization conditions |
| Chemical compound, drug | 2-methyl-1-butene | Sigma Aldrich, 257486 | Reaction Substrate | Starting material for synthesis |
| Chemical compound, drug | Pentane-1,5-diol | Sigma Aldrich, P7703 | Reaction Substrate | Starting material for synthesis |
| Chemical compound, drug | AD-mix-β | Sigma Aldrich, 392766 | Reagent | Chiral catalyst for dihydroxylation |
| Chemical compound, drug | Diisobutyl Aluminium Hydride solution | Sigma Aldrich, 214981 | DiBALH in 1M THF | Reducing agent for reactions |
| Chemical compound, drug | Tert-butyl Lithium | Sigma Aldrich, 186198 | *t-*BuLi, 1.6 M in Pentane | Coupling agent |
| Chemical compound, drug | Bis(cyclopentadienyl)titanium(IV) dichloride | Sigma Aldrich, 234826 | Titanocene dichloride | Organometallic reagent |
| Chemical compound, drug | (+)-(*R,R)-* Diethyltartarate | Sigma Aldrich, 156841 | (+)-DET | Chiral catalyst for Sharpless Epoxidation |
| Chemical compound, drug | Titanium Isopropoxide | Sigma Aldrich, 205273 | Ti(iOPr)4 | Catalyst for Sharpless Epoxidation |
| Chemical compound, drug | Grubb’s Second generation catalyst | Sigma Aldrich, 569747 | Grubbs catalyst M204 | Catalyst for. Ring closing metathesis reaction |
